# Elevation of O-GlcNAc and GFAT expression by nicotine exposure promotes epithelial‐mesenchymal transition and invasion in breast cancer cells

**DOI:** 10.1038/s41419-019-1577-2

**Published:** 2019-04-24

**Authors:** Nana Zhang, Tong Zhu, Kairan Yu, Meiyun Shi, Xue Wang, Lingyan Wang, Tianmiao Huang, Wenli Li, Yubo Liu, Jianing Zhang

**Affiliations:** 1 School of Life Science & Medicine, Dalian University of Technology, Panjin, China; 2 School of Life Science & Biotechnology, Dalian University of Technology, Dalian, China

**Keywords:** Breast cancer, Cell migration, Post-translational modifications

## Abstract

Cigarette smoking has been shown to be a carcinogenic factor in breast cancer. Nicotine (Nic), an active component of tobacco, has been found to induce epithelial-mesenchymal transition (EMT) in breast cancer cells. However, the alterations in protein O-GlcNAcylation in Nic-mediated tumorigenesis and malignization mechanisms are less well studied. Herein, we found that cellular O-GlcNAcylation dramatically increased in human breast cancer cells with EMT activation induced by Nic. Elevated O-GlcNAcylation subsequently promoted Nic-induced EMT activation and increased cell migratory abbility. In addition, we demonstrated that a differentiation factor for the mammary epithelium, CCAAT/enhancer-binding protein B (CEBPB), was involved in Nic-induced hyper-O-GlcNAcylation via transcriptional regulation of the expression of the key enzyme glutamine: fructose-6-phosphate amidotransferase (GFAT) and thus increased the flux through the hexosamine biosynthetic pathway (HBP). Finally, elevated O-GlcNAcylation of the transcriptional repressor C/EBP homologous protein (CHOP) suppressed its heterodimerization with CEBPB and facilitated the DNA-binding activity of CEBPB, further generating positive feedback that enhanced EMT upon Nic stimulation. In conclusion, our results have revealed a new regulatory mechanism involving CEBPB/GFAT-induced hyper-O-GlcNAcylation that plays a key role in EMT and smoking-mediated breast cancer progression.

## Introduction

Cigarette smoking is known to be one of the most important risk factors for several malignancies including breast cancer^1–3^. Evidence has shown that ~10–30% of breast cancers are attributed to tobacco use^4^. Moreover, smoking status is associated not only with tumor incidence but also with the subsequent clinical outcome of breast cancer^5,6^. Nicotine (Nic), a well-known carcinogenic component of cigarettes, exerts its biological function mainly through nicotinic acetylcholine receptors (nAChRs) and influences multiple signaling pathways in cancer cells^7^. Research in this area has revealed that Nic induces epithelial-mesenchymal transition (EMT) and increases the aggressiveness of breast cancer cells^8,9^. However, the mechanisms linking smoking to the development of breast cancer are not completely clear.

A plethora of experimental data demonstrate that EMT is an essential process during breast cancer metastasis, which is the leading cause of breast cancer-related death^10^. In the multistep cascade of metastasis development, the function of EMT is associated with the initial events, in which cancer cells lose their epithelial properties to acquire a mesenchymal phenotype and become motile and invasive^11,12^. A full understanding of this highly complex process is therefore critical for developing next-generation therapies. Intricate circuits involving cooperation between signaling pathways, transcriptional regulation and posttranslational regulation also seem to govern EMT^13^. Emerging evidence suggests that a posttranslational modification of O-linked *N*-acetylglucosamine (O-GlcNAc) in cancers may be involved in EMT activation^14^. O-GlcNAcylation is a highly dynamic form of glycosylation in which the monosaccharide *N*-acetylglucosamine is attached to serine/threonine residues of a wide variety of cytosolic and nuclear proteins by the enzymes O-GlcNAc transferase (OGT) and O-GlcNAcase (OGA)^15^. Elevated O-GlcNAcylation of EMT-related regulators E-cadherin, Vimentin, β-catenin, and Snail has been reported to influence their expression and/or DNA-binding activity^16,17^. Therefore, hyper-O-GlcNAcylation is linked to the enhancement of invasion and metastasis in various tumor types, including breast cancer.

Analogous to phosphorylation, the levels of O-GlcNAcylation are dynamically elevated in response to diverse forms of cellular stress and stimulation, including DNA damage, ER stress and drug treatment, to promote cell homeostasis and survival^18–20^. In addition to OGT dysregulation, the increase in cellular UDP-GlcNAc resulting from the flux though the hexosamine biosynthetic pathway (HBP) also drives hyper-O-GlcNAcylation^21^. The HBP accounts for ~2–5% of the total glucose that enters the cell and provides UDP-GlcNAc as the monosaccharide donor molecule for O-GlcNAcylation^20^. The limiting step of the HBP is catalyzed by glutamine: fructose-6-phosphate amidotransferase (GFAT), which is dysregulated in breast cancer and associated with tumor progression and relapse^17,18^. Thus, the GFAT-governed HBP flux might directly influence Nic-induced EMT in breast cancer cells by altering cellular O-GlcNAcylation. However, the effect and regulatory mechanism(s) of Nic related to O-GlcNAcylation and its contribution to breast cancer metastasis remain largely unknown.

In this study, we present evidence that Nic elevates cellular O-GlcNAcylation through inducing GFAT expression and amplifying the HBP flux, resulting in the promotion of EMT in breast cancer cells. The EMT- and mammary epithelium differentiation-related transcription factor CCAAT/enhancer-binding protein B (CEBPB) was demonstrated to play a critical role in Nic-induced GFAT transcription. Furthermore, O-GlcNAcylation of the transcriptional repressor C/EBP homologous protein (CHOP) suppressed its binding to CEBPB and generated positive feedback that enhanced GFAT expression and EMT activation in breast cancer cells. In summary, we have identified a previously unappreciated regulatory mechanism of GFAT wherein it increases cellular O-GlcNAcylation, which then accounts for smoking-mediated breast cancer progression.

## Materials and methods

### Cell culture and reagents

MCF-7, T47D, MDA-MB-435, and MDA-MB-231 cells were obtained from Type Culture Collection of the Chinese Academy of Sciences (Shanghai, China) and were used within 6 months from resuscitation. All the cells were cultured in 90 % RPMI-1640 (Gibco, USA) supplemented with 1% penicillin/streptomycin antibiotics (Gibco, USA) and 10% fetal bovine serum (FBS, Gibco, USA). Nicotine (Nic, Sigma, MO, USA) was added to the cells cultured in indicated concentrations. Azaserine (AZA), PugNAc were purchased from Sigma (MO, USA). **L01** was purchased from BioBioPha Co., Ltd. (Kunming, China). Lipofectamine 2000 was from Invitrogen (NY, USA). O-GlcNAc enzymatic labeling system were from Invitrogen (CA, USA). Human CEBPB and CHOP cDNA were respectively subcloned into the pcDNA3.1 mammalian expression vector (Invitrogen, NY, USA). GFAT promoter report constructs were made by cloning wild type 2384 bp of human GFAT promoter (WT-Luc) or mutants (MUT-Luc, GATTACTCCAC → GAAAACTCCAC; ATTACACAAG → AAAACACAAG) into luciferase vector pGL3. Primers were shown in Supporting information Table S1.

### Quantitative RT-PCR

Total RNA was isolated using the Trizol method (Invitrogen, NY, USA). A total of 5 μg of RNA were reverse-transcribed and amplified using One Step SYBR PrimeScript PLUS RT-PCR Kit (TaKaRa, Dalian, China) and the Thermal Cycler Dice instrument (TaKaRa, Dalian, China) according to the manufacturer’s instructions. RT-PCR primers are listed in Table S1. Results were normalized to GAPDH.

### Cell migration assay

For wound healing assay, briefly, cells were seeded in six-well plates and scratched by a 200-μL tip to create a wound. Cells were then incubated with Nic in RPMI-1640/0.7% FBS for 48 h and photographed under an inverted microscope. In order to determine the percentage scratch closure, the cell free space was measured using cellSens software and pixels were converted to micrometers (*n* = 6 from four independent isolation procedures). The migration value was calculated as wound closure distance of different group respectively.

For the cell invasion assay, the chamber inserts of 24-well transwell plate (Corning, CA, USA) was used to assess the invasion capacities of cells. Melting ECMatrix gel (BD, MA, USA) overnight at 4 °C, coated chamber according to the manufacturer’s protocol. Cells (6 × 10^4^) were transient transfected for 24 h. Cells were pre-treated with 5 mM mitomycin-C (Sigma, MA, USA) for 12 h to inhibit proliferation, then seeded in the upper chamber. 12 h later, cells had invaded through the ECMatrix gel and finally counted with microscope of ×400 magnification. The independent experiments were run in three times.

For the cell migration assay, cells (6 × 10^4^) were transient transfected for 24 h. Cells were pre-treated with 5 mM mitomycin-C for 2 h to inhibit proliferation, then cells were seeded in the upper chamber of transwell plate filled with serum-free medium, while the lower chamber contained complete culture medium supplemented with 10% FBS. Four hours later, the cells that had passed through the upper chamber was counted. The experiments were repeated three times.

### Immunocytochemistry

Cells were fixed in 4% paraformaldehyde at 37 °C for 15 min and washed 3 times with PBS. Cells were permeabilized using 0.1% Triton X-100 in PBS-T for 0.5 h and blocked with 5% goat serum in 1% bovine serum for 1 h at room temperature. Then the cells were incubated with primary antibodies diluted in 5% goat serum in 1% bovine serum overnight at 4 °C. Secondary antibody were used to visualize the proteins. Cells were coverslipped with Vectashield Mounting Medium with DAPI (Thermo, MA, USA) and mounted onto slides. Image acquisition was performed on a Leica confocal microscope (Leica, CA, USA).

### Immunoblot and immunoprecipitation

The cell lysing, western blotting and immunoprecipitation were performed as previously described^22^. The following antibodies were used: OGT, OGA, GAPDH, XBP1, GFAT, CHOP, CEBPB, Snail, pSnail (S246), Vimentin, Twist, N-cadherin and E-cadherin were from Abcam (MA, USA); O-GlcNAcylation antibody (CTD110.6) was from BioLegend (MA, USA). Chemiluminescent detection was performed using ECL kit (GE healthcare, CA, USA).

### Gene silencing and transfection

Gene silencing was achieved though transfection with siRNA using the Lipofectamine 2000 transfection reagent following the manufacturer’s instructions. OGT siRNA (sc-40780), GFAT siRNA (sc-6068), CEBPB siRNA (sc-29862), CHOP siRNA (sc-156118), XBP1 siRNA (sc-38627) and scrambled siRNA (sc-37007) were obtained from Santa Cruz Biotechnology (CA, USA). Transfection of the MCF-7 and MDA-MB-231 cells were performed with Lipofectamine 2000 according to the manufacturer’s instructions.

### Reporter gene assays

Cells were plated at a density of 1 × 10^4^ cells/well in 96-well culture plates and then transfected as described above. Briefly, indicated cells were transfected with 0.1 mg of GFAT WT-Luc or MUT-Luc. Reporter gene activities were measured with a dual luciferase assay system (Promega, MA, USA) 48 h after transfection. For co-transfection experiments, 0.02 mg of the pcDNA3-CEBPB or pcDNA3-CHOP expression plasmid was included. In addition, the expression vector pcDNA3.1 was used as a negative control to evaluate the effects of CEBPB or CHOP on GFAT WT-Luc.

### ChIP assay

Quantitative ChIP assays were performed using a commercially available Simple ChIP Kit (Cell Signaling Technology, MA, USA) according to the manufacturer’s instructions. The cross-linked chromatin was then sonicated and immunoprecipitated using anti-CHOP and anti-CEBPB antibodies (Cell Signaling Technology, MA, USA). GFAT promotor primers are listed in supplementary material Table S1.

### Statistical analysis

All data are presented as the mean ± standard errors of the mean (SEM), *N* = 3. Data groups were compared by two-tailed Student’s t-test using the GraphPad Software. Differences between groups were considered statistically significant if *p* < 0.05. The statistical significance is denoted by asterisks (*p < 0.05; **p *<* 0.01; ***p *<* 0.001).

## Results

### Nic-induced hyper-O-GlcNAcylation promotes EMT and invasion in breast cancer cells

**Fig. 1:**
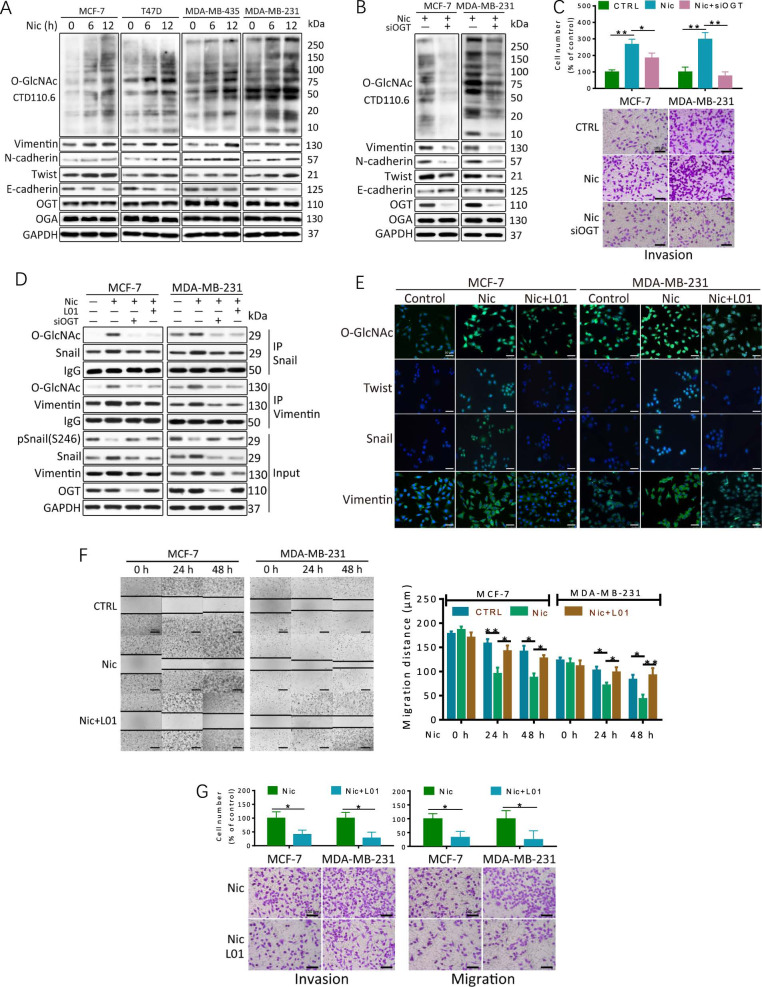
Hyper-O-GlcNAcylation levels contribute to Nic-induced EMT and invasion in breast cancer cells. **a** O-GlcNAcylation was upregulated in breast cancer cells after treatment with Nic. Breast cancer cells were treated with 100 μM Nic for the indicated times. Cellular O-GlcNAcylation and the expression of EMT markers in breast cancer cell lines were analyzed by immunoblotting analysis. (**b**, **c**) MCF-7 and MDA-MB-231 cells were transfected with scrambled siRNA (−) or OGT siRNA (siOGT, +) for 48 h and then treated with 100 μM Nic for an additional 24 h. Protein levels were examined by immunoblotting. Transwell invasion assays showed that the reduction of O-GlcNAcylation inhibited the Nic-induced invasion of breast cancer cells. **d** The EMT-related proteins Snail and Vimentin were O-GlcNAcylated during Nic treatment. MCF-7 and MDA-MB-231 cells were transfected with scrambled siRNA (−) or OGT siRNA (siOGT, +) for 48 h and then treated with 100 μM Nic for 24 h. 100 μM **L01** was used to inhibit O-GlcNAcylation. Snail and Vimentin immunoprecipitations assays were performed, and the immunoprecipitated fractions were analyzed by immunoblotting. **e** Immunocytochemistry of MCF-7 and MDA-MB-231 cells treated with 100 μM Nic either alone or together with 100 μM **L01** for 12 h. The immunoreactivity of the antibodies for O-GlcNAc CTD110.6 and EMT markers is shown in green, and DAPI is shown in blue. **f** Wound healing assays and Transwell migration and invasion assays showed that the reduction of O-GlcNAcylation inhibited the Nic-induced migration and invasion of breast cancer cells. Cells were treated with 100 μM Nic either alone or together with 100 μM **L01** for 24 h and 48 h. Graphs comparing the average migration rate in different treated cells are shown. The data represent the mean ± SEM, *N* = 3, *p < 0.05, **p < 0.01

Given the important role of O-GlcNAcylation in response to cellular stress^17,18^, we first investigated whether the protein O-GlcNAcylation levels contribute to Nic-induced EMT and invasion in breast cancer cells. Immunoblot analysis revealed a dynamic increase in the O-GlcNAcylation of multiple proteins in breast cancer cells (MCF-7, T47D, MDA-MB-435, MDA-MB-231) upon treatment with 100 μM Nic for 6 and 12 h (Fig. 1a). Treatment with 1 μM, 10 μM, or 100 μM Nic induced elevation of cellular O-GlcNAc levels in a dose-dependent manner in both MCF-7 and MDA-MB-231 cells (Supporting information Fig. S1A). Consistent with previous reports, EMT markers, including Vimentin, N-cadherin and Twist, were elevated by Nic, whereas E-cadherin declined (Fig. 1a and Fig. S1A). However, the levels of enzymes that govern the O-GlcNAc metabolism, OGT and OGA, remained constant, indicating that other mechanisms may be involved in the regulation of O-GlcNAcylation. Nic induces similar biochemical changes in other types of smoking-related cancer cells (Fig. S1B). Other nitrosamine tobacco carcinogens, such as 4-(methylnitrosamino)-1-(3-pyridyl)-1-butanone (NNK) and N’-nitrosonornicotine (NNN), could also induce O-GlcNAc elevation and EMT marker upregulation in MDA-MB-231 cells (Fig. S1C).

Further, suppressing Nic-induced dynamic O-GlcNAcylation by OGT silencing decreased the levels of EMT markers and cell invasion ability in Nic-treated MCF-7 and MDA-MB-231 cells (Fig. 1b, c). Notably, treatment with OGT siRNA modestly decreased invasion ability in MCF-7 cells with low nAChR expression levels, whereas it drastically attenuated the invasion of MDA-MB-231 cells with high nAChR expression levels (Fig. 1c and S1D). Moreover, Nic augmented Snail O-GlcNAcylation, which stabilizes Snail1 by suppressing its phosphorylation (S246) and stimulates EMT through transcriptional suppression of E-cadherin (Fig. 1d and S2). Following OGT siRNA treatment, the Snail protein level was reduced, while its phosphorylation (S246) and the E-cadherin level were increased compared with Nic treatment alone in MCF-7 and MDA-MB-231 cells. Similar results were obtained for another O-GlcNAcylated EMT marker, Vimentin (Fig. 1d). Accordingly, the suppression of O-GlcNAcylation by our previously developed OGT inhibitor **L01**^23^ remarkably decreased the expression of Twist, Snail and Vimentin in an immunofluorescence assay (Fig. 1e). Treatment of cells with **L01** also significantly decreased the motility of both MCF-7 and MDA-MB-231 cells under Nic conditions (Fig. 1f).

Together, these data suggest that the upregulation of the protein O-GlcNAcylation levels contributes mechanistically to Nic induced EMT and invasion in breast cancer cells.

### Nic elevates O-GlcNAcylation in breast cancer cells via activation of GFAT and HBP

**Fig. 2:**
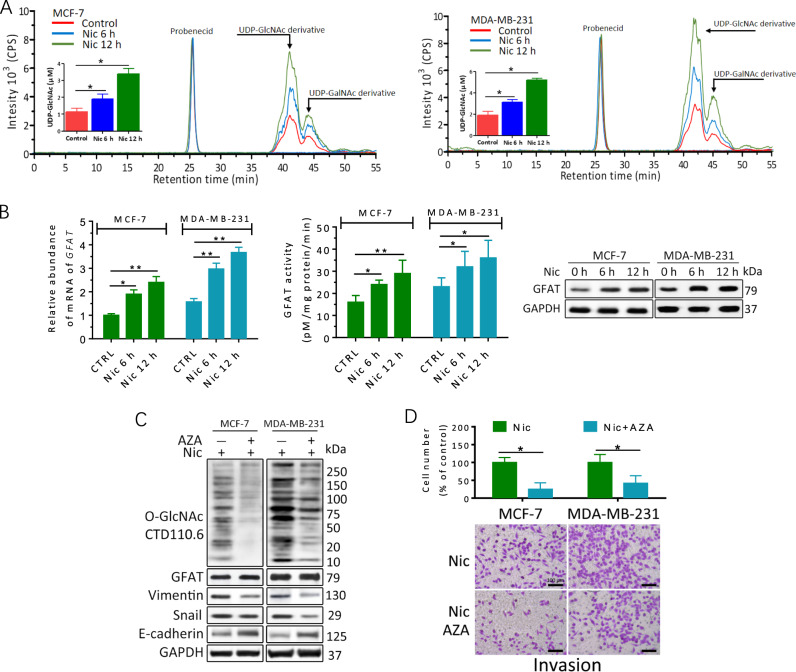
The HBP flux and GFAT are upregulated in breast cancer cells in response to Nic. **a** Nic induced increases in UDP-GlcNAc levels. UDP-GlcNAc in the cell lysates was derivatized with trimethylsilyldiazomethane. Chromatograms of polar metabolites in MCF-7 and MDA-MB-231 cell extracts from control cells (red line) and cells treated with 100 μM Nic for 6 h (blue line) or 12 h (green line) show regions corresponding to the UDP-GlcNAc derivative, UDP-GalNAc derivative and probenecid retention times. Quantitative analyses are shown. Quantitative analyses are shown with the standard deviation based on three independent experiments *P*-values were calculated using one-way ANOVA and the appropriate post test. *P < 0.05. **b** GFAT transcriptional levels, protein levels and activity were upregulated in breast cancer cells after treatment with Nic for the indicated times. The GFAT transcript level was analyzed by quantitative RT-PCR. DMSO was used as a control (CTRL). Whole-cell lysates were analysed by immunoblotting. Cell lysates were also used for the analysis of GFAT activity. **c**, **d** MCF-7 and MDA-MB-231 cells were treated with 100 μM Nic either alone or together with 25 μM AZA for 24 h. Protein levels were examined by immunoblotting. DMSO was used as a control (CTRL). Transwell invasion assays showed that inhibition of GFAT reduced Nic-induced the invasion of breast cancer cells. The data represent the mean ± SEM, *N* = 3, *p < 0.05, **p < 0.01

As hyper-O-GlcNAcylation can be supported by the HBP, the effects of Nic on the levels of the HBP end product UDP-GlcNAc were examined. By using derivatization and mass spectrum (MS) analysis, UDP-GlcNAc was distinguished from UDP-GalNAc in cell lysates (Fig. 2a and S3A). Nic treatment induced remarkable UDP-GlcNAc accumulation in MCF-7 and MDA-MB-231 cell extracts, suggesting metabolic reprogramming of the HBP by Nic stimulation in breast cancer cells. Then, the expression of the key enzyme involved in the synthesis of UDP-GlcNAc, GFAT, was examined. As shown in Fig. 2b and Fig. S1A, increases in GFAT activity and mRNA and protein expression were observed in both breast cancer cell lines when cultured in Nic. The effect of Nic was almost completely blocked by inhibiting α9-nAChR in MCF-7 and MDA-MB-231 cells, while inhibition of α7-nAChR moderately attenuated the Nic-induced increase in GFAT and EMT marker expression (Fig. S1E), suggesting that the effect of Nic is primarily mediated by α9-nAChR subunits. We further analyzed expression data in the GEPIA database (http://gepia.cancer-pku.cn/index.html). The results demonstrated that GFAT expression was upregulated in breast cancer and other smoking-related tumors compared with the normal tissues. GFAT expression was also found to be negatively associated with the overall survival of breast cancer patients (Fig. S3B, C).

To further test the role of GFAT in Nic-induced hyper-O-GlcNAcylation and EMT activation, azaserine (AZA), an inhibitor of GFAT, was applied. Pretreatment with AZA drastically suppressed cellular O-GlcNAcylation and Vimentin expression triggered by Nic (Fig. 2c). In contrast, E-cadherin expression was enhanced by AZA in Nic treated cells. Moreover, inhibition of GFAT significantly abolished the Nic-elicited invasion of MDA-MB-231 cells (Fig. 2d). A modest decrease in GFAT mRNA was also observed (Fig. S3D). Consistent results were observed in MCF-7 cells. These data demonstrated that upregulation of GFAT by Nic is responsible for O-GlcNAc elevation and promotes EMT in breast cancer cells.

### CEBPB mediates the effects of Nic on GFAT in breast cancer cells

**Fig. 3:**
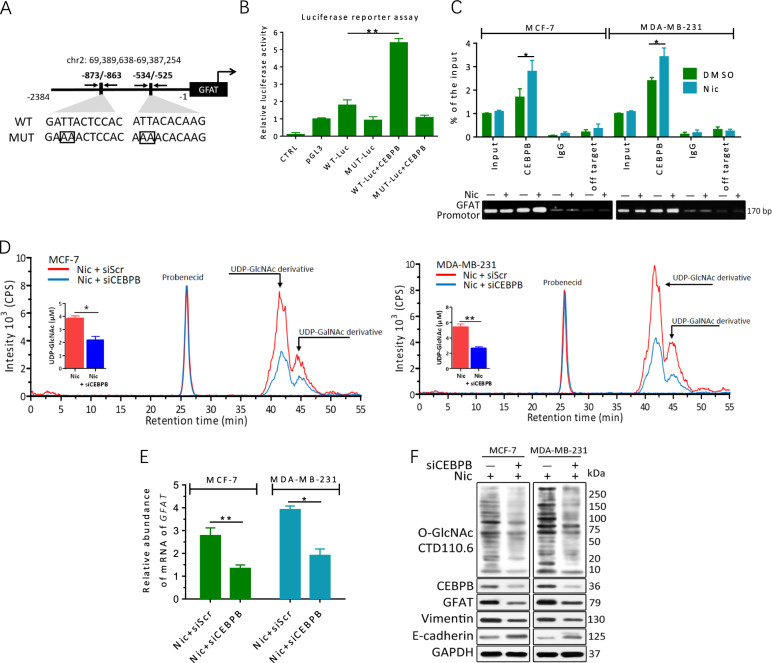
GFAT accumulation upon Nic is regulated by CEBPB. **a** Schematic description of the putative GFAT distal promoter with potential CEBPB-binding sites. A wild-type GFAT promoter (WT) luciferase reporter construct and a mutant (MUT) form lacking the CEBPB-binding site are shown. **b** 293T cells were transfected with a reporter vector consisting of luciferase cDNA fused to the GFAT promoter. The pGL3 vector and mutational (MUT) GFAT promoter were used as controls. Transfection of CEBPB increased GFAT promoter activity. **c** MCF-7 and MDA-MB-231 cells were treated with or without 100 μM Nic for 24 h. The lysates were subjected to ChIP. Quantitative PCR amplification was performed using primers specific for the GFAT promoter. **d** The induction of UDP-GlcNAc by Nic was attenuated by CEBPB inhibition. Chromatograms of polar metabolites in MCF-7 and MDA-MB-231 cell extracts from cells treated with either 100 μM Nic alone (red line) or CEBPB siRNA plus Nic (blue line) for 24 h show regions corresponding to the UDP-GlcNAc derivative, UDP-GalNAc derivative and probenecid retention times. **e** GFAT transcriptional levels were reduced in breast cancer cells after CEBPB knockdown. The GFAT transcript level was analyzed by quantitative RT-PCR. Scrambled siRNA was used as a control. **f** MCF-7 and MDA-MB-231 cells were transfected with scrambled siRNA (-) or OGT siRNA (siCEBPB, +) for 48 h and then treated with 100 μM Nic for an additional 24 h. The protein levels were examined by immunoblotting. The data represent the mean ± SEM, *N* = 3, *p < 0.05, **p < 0.01

To further explore the regulatory mechanism of GFAT accumulation related to Nic stimulation, sequence analysis of the GFAT promoter was performed. In silico analyses revealed that two binding motifs of the EMT- and mammary epithelium differentiation-related transcription factor CEBPB were located 525 bp and 863 bp upstream from the transcription start site of GFAT (Fig. 3a). To identify the potential binding site, we subcloned the proximal wild-type (WT) promoter of human GFAT into a luciferase reporter vector. A double C/EBP mutant (MUT) was used as the negative control for the gene reporter assay. Reporter assays showed that coexpression of CEBPB dramatically increased the luciferase activity of the WT promoter without affecting MUT activity in 293T cells (Fig. 3b), suggesting that CEBPB directly stimulates the transcription of GFAT.

To corroborate this finding in breast cancer cells, we performed chromatin immunoprecipitation (ChIP) analysis in MCF-7 and MDA-MB-231 cells. Random primers that could not specifically bind the GFAT promoter region were used as an off-target negative control. RT-PCR and qPCR showed enrichment of the GFAT promoter region in the CEBPB precipitate (Fig. 3c). In addition, the binding of CEBPB to the C/EBP element in the GFAT promoter was significantly increased after the cells were treated with Nic. Silencing of CEBPB with siRNA obviously impeded Nic-induced GFAT transcription, UDP-GlcNAc accumulation and hyper-O-GlcNAcylation in MCF-7 and MDA-MB-231 cells (Fig. 3d, e). In contrast, siXBP1 did not significantly reduce Nic-induced GFAT transcription (Fig. S4). CEBPB knockdown also attenuated the triggering of EMT activation by Nic through suppressing Vimentin expression and robustly upregulating E-cadherin levels (Fig. 3f). Collectively, our results reveal that CEBPB activates endogenous GFAT expression and increases cellular O-GlcNAcylation in Nic-treated breast cancer cells.

### O-GlcNAcylation enhances CEBPB-mediated GFAT transcription in Nic-treated cells

**Fig. 4:**
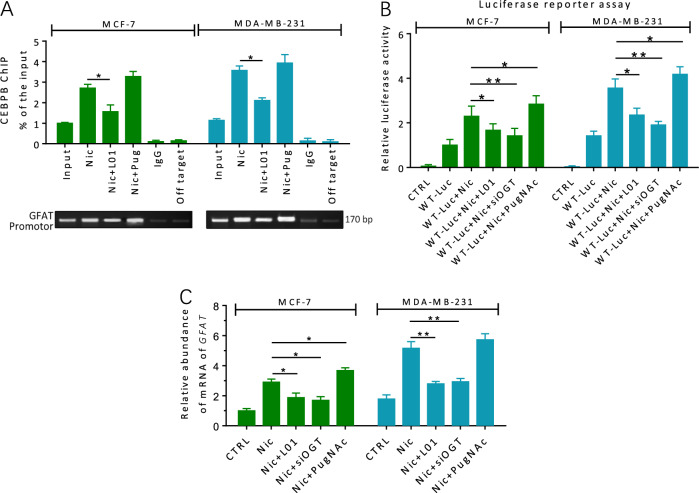
O-GlcNAcylation is involved in the control of GFAT transcription in Nic-treated breast cancer cells. **a** MCF-7 and MDA-MB-231 cells were treated with 100 μM Nic either alone or together with **L01** (100 μM)/PugNAc (50 μM) for 24 h. The lysates were subjected to ChIP assays. Quantitative PCR amplification was performed using primers specific for GFAT promoter. **b** Indicated cells were transfected with a reporter vector consisting of luciferase cDNA fused to GFAT promotor. Transfection of OGT siRNA or inhibition of O-GlcNAc (100 μM **L01**) decreased GFAT promotor activity. PugNAc (50 μM) increased GFAT promotor activity. **c** O-GlcNAcylation regulates GFAT transcription in breast cancer cells with Nic treatment (100 μM, 24 h). Transfection of OGT siRNA or inhibition of O-GlcNAc (100 μM **L01**) decreased GFAT transcription. PugNAc (50 μM) increased GFAT transcription. GFAT transcript level was analyzed by quantitative RT-PCR. Scrambled siRNA was used as a control (CTRL). The data represent the mean ± SEM, *N* = 3, *p < 0.05, **p < 0.01

To assess the functional significance of O-GlcNAcylation in Nic-induced GFAT activation, ChIP analysis was performed to test whether cellular O-GlcNAcylation status affected the DNA-binding activity of CEBPB. MCF-7 and MDA-MB-231 cells were treated with/without **L01** or the OGA inhibitor PugNAc for 24 h under Nic conditions (Fig. 4a). The ChIP results showed that the binding of CEBPB to the GFAT promoter was obviously decreased after cellular O-GlcNAcylation was suppressed by **L01**. On the other hand, treatment with the PugNAc, which increased O-GlcNAcylation, enhanced the DNA-binding activity of CEBPB. We then performed a luciferase reporter assay for the GFAT promoter and investigated endogenous GFAT mRNA levels in MCF-7 and MDA-MB-231 cells (Fig. 4b). Both the OGT inhibitor **L01** and the OGT siRNA used in our experiments resulted in the suppression of GFAT promoter activity and GFAT mRNA transcription compared with Nic treatment alone, while the opposite effect was observed when PugNAc was used (Fig. 4c). Together, these data strongly suggest that O-GlcNAcylation is involved in the control of GFAT transcription and further generates positive feedback that strengthens EMT upon Nic stimulation in breast cancer cells.

### O-GlcNAcylation suppresses the binding of the negative regulator CHOP to CEBPB

**Fig. 5:**
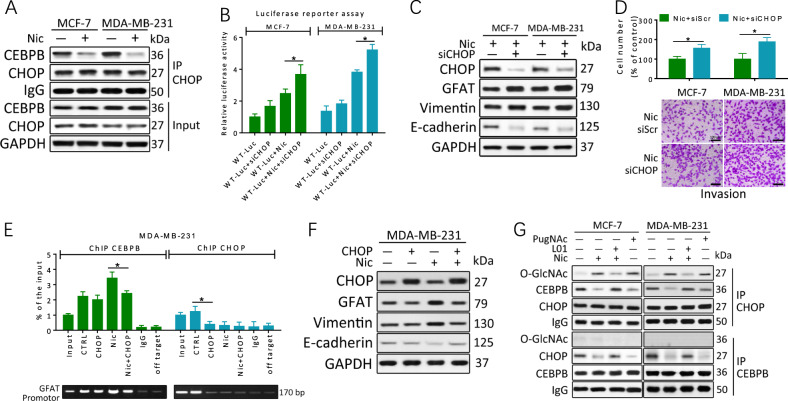
O-GlcNAcylation of CHOP suppresses its transcriptional inhibition of CEBPB. **a** CHOP/CEBPB dimer is reduced in breast cancer cells with Nic treatment (100 μM, 24 h). CHOP co-IP were performed, and immunoprecipitated fractions were analyzed by immunoblotting for the indicated proteins. **b** Indicated cells were transfected with a reporter vector consisting of luciferase cDNA fused to GFAT promoter and then treated for 24 h. CHOP knockdown with siRNA (siCHOP) increased GFAT promoter activity. **c**, **d** MCF-7 and MDA-MB-231 cells were transfected with scrambled siRNA (−) or CHOP siRNA (siCHOP, +) for 48 h and then treated with 100 μM Nic for an additional 24 h. Protein levels were examined by immunoblotting. Transwell invasion assays showed that reduction of O-GlcNAcylation inhibited Nic-induced invasion in breast cancer cells. **e**, **f** MDA-MB-231 cells were treated with 100 μM Nic either alone or together with transfection of pcDNA3-CHOP for 24 h. pcDNA3.1 vector was used as a control (CTRL). The lysates were subjected to ChIP assays with indicated antibodies. Quantitative PCR amplification was performed using primers specific for GFAT promoter. The protein levels were examined by immunoblotting. **g** O-GlcNAc modification of CHOP is an obstacle for transcriptional inhibition of CEBPB. MCF-7 and MDA-MB-231 cells were treated with 100 μM Nic either alone or together with **L01** (100 μM)/PugNAc (50 μM) for 24 h. Immunoprecipitation was performed, and immunoprecipitated fractions were analyzed by immunoblotting for the indicated proteins. The data represent the mean±SEM, *N* = 3, *p < 0.05

Previous studies have shown that transcription mediated by CEBPB is influenced by its heterodimerization with ER stress-related CHOP^24^. Co-IP experiments confirmed that CHOP was associated with CEBPB in MCF-7 and MDA-MB-231 cells. However, the amount of this dimer was significantly reduced under Nic conditions, while CHOP and CEBPB levels were constant (Fig. 5a). In comparison with GFAT promotor WT-Luc transfected MCF-7 and MDA-MB-231 cells in luciferase reporter assays, CHOP knockdown cells showed a marked increase in GFAT promoter activity with Nic treatment (Fig. 5b). The downregulation of CHOP by siRNA also enhanced GFAT expression under Nic conditions but led to only a modest upregulation of GFAT in the absence of Nic (Fig. 5c). Moreover, CHOP downregulation stimulated EMT activation and enhanced invasion (Fig. 5d). Subsequent ChIP assays showed that the putative CEBPB-binding region in the endogenous GFAT promoter was specifically present in anti-CEBPB, but not anti-CHOP, immunoprecipitates from MDA-MB-231 cells treated with Nic (Fig. 5e). Overexpression of CHOP prevented the DNA binding of CEBPB and decreased the levels of GFAT as well as the EMT markers in Nic-treated MDA-MB-231 cells (Fig. 5e, f), suggesting the crucial role of CHOP in the Nic-induced upregulation of GFAT.

In addition, CHOP, but not CEBPB, was found to be O-GlcNAcylated during Nic treatment (Fig. 5g). Endogenous CHOP was specifically enriched in Nic-treated MCF-7 and MDA-MB-231 cells, followed by detection with the O-GlcNAcylation antibody CTD110.6. O-GlcNAcylated CHOP was further detected by a complementary approach using sWGA (Fig. S5). To exclude the possibility of nonspecific recognition by WGA and/or CTD110.6, a commercially available enzymatic method (the O-GlcNAc Enzymatic Labeling System) that involves chemo-selective ligation of biotin to terminal GlcNAc residues was used to confirm this result (Fig. S6). We were able to unequivocally detect O-GlcNAc on CHOP in an amount that could be significantly decreased by OGT inhibition. In contrast, invisible O-GlcNAcylated CHOP was detected in the control breast cancer cells. Further, **L01** treatment increased the amount of CHOP coimmunoprecipitated with CEBPB (Fig. 5g) in Nic treated breast cancer cells. By contrast, PugNAc reduced the interaction with CHOP relative to CEBPB in breast cancer cells without Nic stimulation, indicating that O-GlcNAc modification of CHOP could represent an obstacle to its interaction with CEBPB. Therefore, the O-GlcNAc modification of CHOP is important for its transcriptional inhibition of CEBPB as well as for Nic-induced and GFAT-dependent EMT activation in breast cancer cells.

## Discussion

**Fig. 6:**
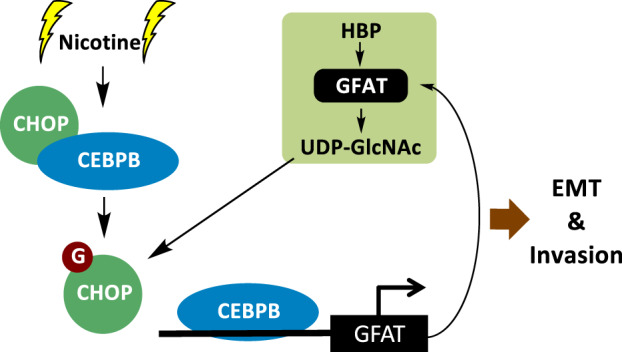
Proposed model for EMT and invasion signaling based on Nic-induced activation of O-GlcNAcylation. Nic stimulates CEBPB-dependent transcription of GFAT and then mediates the production of UDP-GlcNAc and hyper-O-GlcNAcylation. Excess O-GlcNAcylation promotes the activation of EMT and breast cancer cell invasion. The O-GlcNAcylation of CHOP also prevents its inhibition of CEBPB, subsequently enhancing GFAT transcription in a positive feedback manner

The effect of cigarette smoking on various types of human cancers has been widely reported^1,25^. Tobacco carcinogens such as Nic not only play a role in the initiation of cancer but also increase the aggressiveness of existing cancers by enhancing growth, motility and metastasis^26,27^. It has been reported that Nic in the bloodstream can be transported to mammary tissue through plasma lipoproteins. Furthermore, because of the lipophilicity of Nic, it may be stored in breast adipose tissue and metabolized and activated by mammary epithelial cells^28^. Although the effect of Nic on cancer cells has been studied, the association of Nic-induced aberrant glycosylation with breast cancer pathogenesis is unknown. Our present study showed for the first time that the activation of O-GlcNAcylation by Nic is especially responsible for the promotion of breast cancer. Nic mediates production of UDP-GlcNAc and hyper-O-GlcNAcylation through CEBPB-dependent activation of GFAT and HBP flux, which further promotes EMT and motility in breast cancer. In this paper, we also demonstrated that the suppressor of CEBPB, CHOP is modified by O-GlcNAc. O-GlcNAcylation prevents the binding of CHOP to CEBPB, subsequently strengthening the DNA-binding activity of the latter in a positive feedback manner (Fig. 6).

Nic exerts its biological effect by binding with nAChRs, which play regulatory roles in various biological processes including cell growth, differentiation, and motility^8^. In breast cancer, nAChRs are reported to act as a key mediator in Nic-induced metastasis^29^. Our data showed that the Nic-induced motility of MDA-MB-231 cells with high expression of α7/α9-nAChRs was dramatically reduced by O-GlcNAc inhibition, whereas O-GlcNAc inhibition slightly suppressed the migratory ability of MCF-7 cells with lower levels of α7/α9-nAChRs. Further, we proved that α9-nAChR, not α7-nAChR, was primarily associated with Nic-induced hyper-O-GlcNAcylation in breast cancer cells. Previous studies have reported that nAChR participates in tumor EMT, which is an essential event during tumor metastasis in many types of cancer^30^. However, the mechanisms of EMT activation in smoking-mediated breast cancer are not completely clear. Cellular O-GlcNAcylation was dynamically upregulated upon Nic treatment, suggesting that abnormalities in O-GlcNAcylation participate in Nic-induced EMT process. A growing body of evidence suggests an important role of O-GlcNAcylation in modulating cell plasticity during EMT^14,15^. In agreement with previous studies, we found that Nic elevated Snail and Vimentin O-GlcNAcylation, which could stabilize these EMT regulators and increase breast cancer cell invasion. The O-GlcNAcylation of Snail, the repressor of E-cadherin transcription, led to the loss of E-cadherin, which is a key step in the initiation of EMT. Notably, inhibition of O-GlcNAcylation showed the opposite effect and reduced cell invasion induced by Nic. This is a noteworthy finding, since O-GlcNAcylation has been proved to be an important regulator of smoking-mediated cancer progression. Therefore, targeting O-GlcNAcylation specifically in smoking-mediated cancer may be a novel strategy in cancer therapy.

As NNK, and NNN can bind nAChRs with greater affinity than Nic, our findings suggested that both Nic and nitrosamines tobacco carcinogens stimulated breast cancer progression through O-GlcNAc elevation. It has been reported that Nic intake among cigarette-only, E-cigarette and Nic-replacement therapy users is roughly similar^31^. E-cigarette and Nic-replacement therapy users were found to exhibit levels of total Nic equivalents that were at least as high as those in combustible cigarette-only users in an adjusted analysis. On the other hand, our results suggest that although the E-cigarette and Nic-replacement therapy users present significantly lower metabolite levels of tobacco-specific N-nitrosamines than combustible cigarette-only users, long-term E-cigarette and Nic-replacement therapy to maintain the cessation of combustible cigarettes might also confer an increased risk of cancer.

To determine the complexity of Nic-induced O-GlcNAcylation, the changes in the pool of the activated substrates UDP-GlcNAc were analyzed. A significant increase in cellular UDP-GlcNAc was found in Nic-treated breast cancer cells, whereas no detectable change in OGT or OGA expression was found, suggesting the potential for an increased the HBP flux. Obvious increases in the GFAT mRNA and protein levels were detected upon Nic treatment, supporting our hypothesis of HBP activation during Nic stimulation. UDP-GlcNAc serves as a donor substrate in multiple glycosylation reactions, including O-GlcNAcylation. In this study, OGT siRNA and the O-GlcNAc inhibitor **L01** decreased cellular O-GlcNAcylation without affecting other types of glycosylation and then significantly attenuated the Nic-induced invasive phenotype in breast cancer cells, indicating that O-GlcNAcylation, but not other types of glycosylation, plays a key role in nicotine- induced breast cancer cell invasion. Alteration of GFAT expression has been reported in several cancers and has been shown to be associated with tumor progression. However, increased GFAT transcription in response to variable stresses isf rarely reported. As a key regulator of HBP flux, GFAT protein levels are reported to be elevated during EMT. In the present study, our data indicated that the mRNA and protein levels of GFAT were upregulated in response to Nic. Furthermore, the inhibition of GFAT by AZA significantly reduced Nic-induced EMT activation and cell motility, suggesting that GFAT plays a key role in smoking-mediated breast cancer metastasis.

Previous reports indicated that the transcription factor XBP1 binds to the endogenous GFAT promoter and promotes its transcription^22^. In this study, our findings suggested a key role of CEBPB, rather than XBP1, in the upregulation of GFAT expression in response to Nic. CEBPB belongs to the CCAAT/enhancer-binding proteins, which are defined as basic leucine zipper transcription factors and play a role in mammary epithelium differentiation. Emerging evidence has shown that dysregulation of CEBPB is markedly correlated with the malignancy of several tumors, including breast cancer^32^. Moreover, CEBPB has also been found to be an EMT modulator^33,34^. Our results clearly showed that CEBPB could directly bind to the GFAT promoter. Since online analysis of the human GFAT promoter sequence indicates the presence of binding sites for both XBP1 and CEBPB, it is possible that CEBPB is induced by Nic in a cell-type- and context-dependent manner. Moreover, although CEBPB was not found to be modified by O-GlcNAc in Nic-treated breast cancer cells, we demonstrated that O-GlcNAcylation is required for the acquisition of the DNA-binding activity of CEBPB. However, the data reported here revealed that the CEBPB itself could not be modified by O-GlcNAc during Nic stress, indicating that other mechanisms are involved in the regulation of CEBPB transcriptional activation.

CHOP is a member of the C/EBP family and plays a role in energy metabolism, cell proliferation and differentiation^35^. CHOP exhibits deficiency in its DNA-binding domain and lacks the ability to bind the consensus C/EBP sites, and it requires heterodimerization with other family members to play a role as a dominant-negative inhibitor of C/EBPs^36^. Here, we discovered a novel regulatory mechanism of CHOP by O-GlcNAcylation. CHOP was shown to be O-GlcNAcylated during Nic stress, and the increase inf O-GlcNAcylation significantly decreased the binding of CHOP to CEBPB, subsequently increasing the DNA-binding activity of CEBPB to the GFAT promoter in breast cancer cells. These findings potentially establish a positive feedback loop of O-GlcNAcylation and demonstrate that inappropriate O-GlcNAcylation is not merely an effect of Nic-induced carcinogenesis but is a pathologically activated pathway.

In summary, we showed that O-GlcNAcylation was increased under Nic stress in breast cancer cells. Nic stimulation increased O-GlcNAcylated CHOP and suppressed the interaction of CHOP with CEBPB, which augmented the binding of CEBPB to the promoter region of GFAT and directly trans-activated GFAT expression. Elevated O-GlcNAcylation subsequently promoted breast cancer cell EMT and invasion. Our work extends the function of O-GlcNAcylation in modulating smoking-mediated breast cancer progression.

## Supplementary information

Revised Supporting information
