# Correction to: Elevation of O-GlcNAc and GFAT expression by nicotine exposure promotes epithelial-mesenchymal transition and invasion in breast cancer cells

**DOI:** 10.1038/s41419-024-06900-6

**Published:** 2024-08-22

**Authors:** Nana Zhang, Tong Zhu, Kairan Yu, Meiyun Shi, Xue Wang, Lingyan Wang, Tianmiao Huang, Wenli Li, Yubo Liu, Jianing Zhang

**Affiliations:** 1 School of Life Science & Medicine, Dalian University of Technology, Panjin, China; 2 School of Life Science & Biotechnology, Dalian University of Technology, Dalian, China

Correction to: *Cell Death & Disease* 10.1038/s41419-019-1577-2, published online 24 April 2019

The authors regret that in the original article, there were mistakes in Fig. 1G as published. In the first published version of this manuscript, the transwell microscope images presented in Fig. 1G of the MCF-7 Nic L01 group (invasion), MDA-MB-231 Nic group (migration), MDA-MB-231 Nic L01 group (migration) were accidentally misused during the assembly of the figures. This error has been corrected, and Fig. 1G has been updated with a new image, provided below.

The scientific conclusions of our study are not affected by this inadvertent error. All authors agree with this revision request. The authors apologize for any confusion they may have caused.


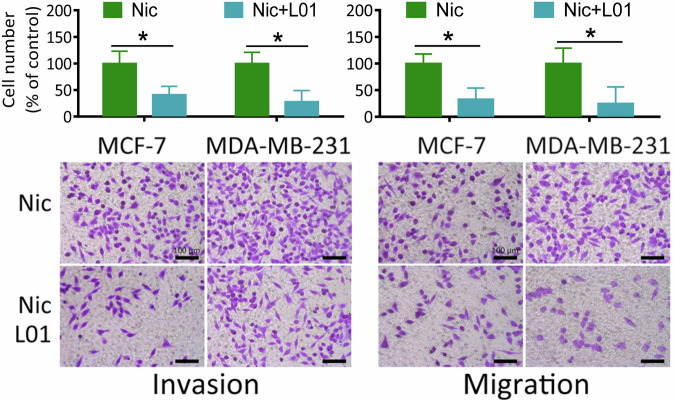


Fig. 1 (F and G) Wound healing assays and Transwell migration and invasion assays showed that the reduction of O-GlcNAcylation inhibited the Nic-induced migration and invasion of breast cancer cells. Cells were treated with 100 μM Nic either alone or together with 100 μM L01 for 24 h and 48 h. Graphs comparing the average migration rate in different treated cells are shown. The data represent the mean ± SEM, *N* = 3, *p < 0.05, **p < 0.01.

The original article has been corrected.

