# Supplementary material for: Elevation of O-GlcNAc and GFAT expression by nicotine exposure promotes epithelial‐mesenchymal transition and invasion in breast cancer cells
Source: Cell Death Dis. 2019 Apr 24;10(5):343. doi: 10.1038/s41419-019-1577-2 (PMC6482138; doi:10.1038/s41419-019-1577-2)
Supplement: Supplementary file 1 — Revised Supporting information [file 41419_2019_1577_MOESM1_ESM.docx]

Supporting information

**Materials and methods**

*Cell culture and reagents*

A549, NCI-H446 and PC-3 cells were obtained from Type Culture Collection of the Chinese Academy of Sciences (Shanghai, China) and were used within 6 months from resuscitation. All the cells were cultured in 90 % RPMI-1640 (Gibco, USA) supplemented with 1 % penicillin/streptomycin antibiotics (Gibco, USA) and 10 % fetal bovine serum (FBS, Gibco, USA). 4-(methylnitrosamino)-1-(3-pyridyl)-1-butanone (NNK), N'-nitrosonornicotine (NNN), α-Bungarotoxin and methyllycaconitine were purchased from Sigma (MO, USA).

*SWGA affinity precipitation*

For sWGA affinity precipitation, cells were lysed with lysis buffer (150mM NaCl, 50mM, Tris, pH 7.4, 1mM EDTA, 0.5% Nonidet P-40) and cell lysates were incubated with agarose-conjugated sWGA (Vector Laboratories, Burlingame, CA) for 3 h. Precipitates were washed four times with lysis buffer and proteins were eluted by boiling in SDS sample buffer.

*In vitro O-GlcNAc assay, enzymatic labelling of CHOP*

CHOP protein was immunoprecipitation with appropriate antibody. To enzymatic label CHOP at O-GlcNAc site, in vitro O-GlcNAcylated CHOP was labeling with Click-iT^™^ O-GlcNAc Enzymatic Labeling System (Invitrogen, NY, USA) and detected by Click-iT^™^ Biotin Protein Analysis Detection Kit (Invitrogen) following manufacturer's recommendation.

*Liquid chromatography-tandem MS (LC-MS/MS) for UDP-GlcNAc quantification.*

UDP-GlcNAc and its chiral isomer UDP-GalNAc were derivatizated as previously described [^1^](#_ENREF_1). Briefly, a 3.0-mL aliquot of trimethylsilyldiazomethane was added to 9.75 mL of methanol: water (3:0.25; v/v) under nitrogen, mixed immediately, and stored under nitrogen in dark conditions. Cells were subjected to ultrasonication in methanol: water (12:1; v/v), the homogenate was then centrifuged. The supernatants containing the UDP-GlcNAc were collected. Then 100 μL supernatant, 10 μL internal standard probenecid (100 ng/mL) and 100 μL derivatization reagent were added to glass tubes, and reacted for 30 min and dried at 40 °C under a stream of nitrogen. The residues were reconstituted in 500 μL of the mobile phase, and 10 μL of the sample was injected into the LC-MS/MS system for analysis. The LC-MS/MS system comprised an UPLC system (Waters Corporation, MA, USA) and Qtrap 6500 mass spectrometer (SCIEX, Toronto, Canada) equipped with an electrospary ionization source in the negative ion mode. A Cosmosil NH_2_ MS column (250 mm×4.6 mm i.d., 5 μm; Cosmosil 5NH_2_-MS, Nacalai Tesque Inc. , Japan) was used. The mobile phase was composed of 40% A (1 mM ammonium acetate) and 60% B (acetonitrile with 1 mM ammonium acetate). The ion spray voltage was adjusted to 4500 V, and the source temperature was set at 500 °C. Multiple reaction monitoring scan mode was used to monitor transitions at m/z 648.0→296.0 for UDP-GlcNAc derivative and UDP-GalNAc derivative, and m/z 283.9→239.9 for internal standard probenecid derivative. The declustering potentials (V) for UDP-GlcNAc, UDP-GalNAc and probenecid were -60, -60 and -50, respectively; and collision energies (eV) were -40, -40 and -20, respectively. Data acquisition and integration were controlled by Applied Biosystems Analyst version 1.6 software. As a result, the retention time for UDP-GlcNAc derivative, UDP-GalNAc derivative and probenecid were 41.5 min, 43.5 min and 26.5 min, respectively. For UDP-GlcNAc quantification, a calibration curve was constructed by analyzing a series of UDP-GlcNAc derivative dilutions spiked with 10 μM Probenecid according to the aforementioned chromatographic method.

*GFAT enzyme activity*

GFAT activity was measured as described previously [^2^](#_ENREF_2). Cells were lysed using extraction buffer (60 mM KH_2_PO_4_, pH 7.0, 1 mM EDTA, and 1 mM dithiothreitol) at 4 °C. The assay mixture contained 15 mM D-fructose-6-phosphate and 15 mM L-glutamine in extraction buffer with 2 mg/mL protein extract. The mixture was incubated at 37 °C for 1 h, and the reaction was terminated by heating at 100 °C for 2 min. After cooling and centrifugation, the end-product D-glucosamine-6-phosphate was estimated through derivatization by ortho-phthalaldehyde and as a fluorimetry read-out. GFAT activity is represented as pmol/mg protein/min.

Table S1 Primers of quantitative RT-PCR

| Gene | Sense | Antisense |
| --- | --- | --- |
| E-cadherin | 5’-GTCATCCAACGGGAATGCA-3’ | 5’-TGATCGGTTACCGTGATCAAAA-3’ |
| GFAT | 5’-AACTACCATGTTCCTCGAACGA-3’ | 5’-CTCCATCAAATCCCACACCAG-3’ |
| GADPH | 5’-TGGTGAAGCAGGCATCTGAG-3’ | 5’-CTCCTGCGACTTCAACAGCA-3’ |
| α7 nAChR | 5’-TCCCCGGCAAGAGGAGTGAA-3’ | 5’-GAGGGCGGAGATGAGCACAC-3’ |
| α9 nAChR | 5’-ATGCACCGGCCATCACCAAA-3’ | 5’-GATCTCCGCTGTCCAAGGCG-3’ |
| GFAT promotor | 5’- TTCTTCTCCTTCAGGCCTTATTC-3’ | 5’-AACCATGCGCCAGTTAAGA-3’ |
| GFAT promotor- CEBPB ChIP-qPCR | 5’-GAGCCTTACATTCCTCCTCTAC -3’  5’-AGGGTTCTGAGGCTAGATGTAT-3’ | 5’-GGTCAGCCTCTCTGGTAATAAG-3’  5’-GGATCACCCAGAGCAAAGTT-3’ |
| ChIP off-target negative control | 5’GGCCACGCGTCGACTAGTCAGA-3’ | 5′-CATCCGGGGTCAGCACCGTTTC-3′ |

Fig.S1


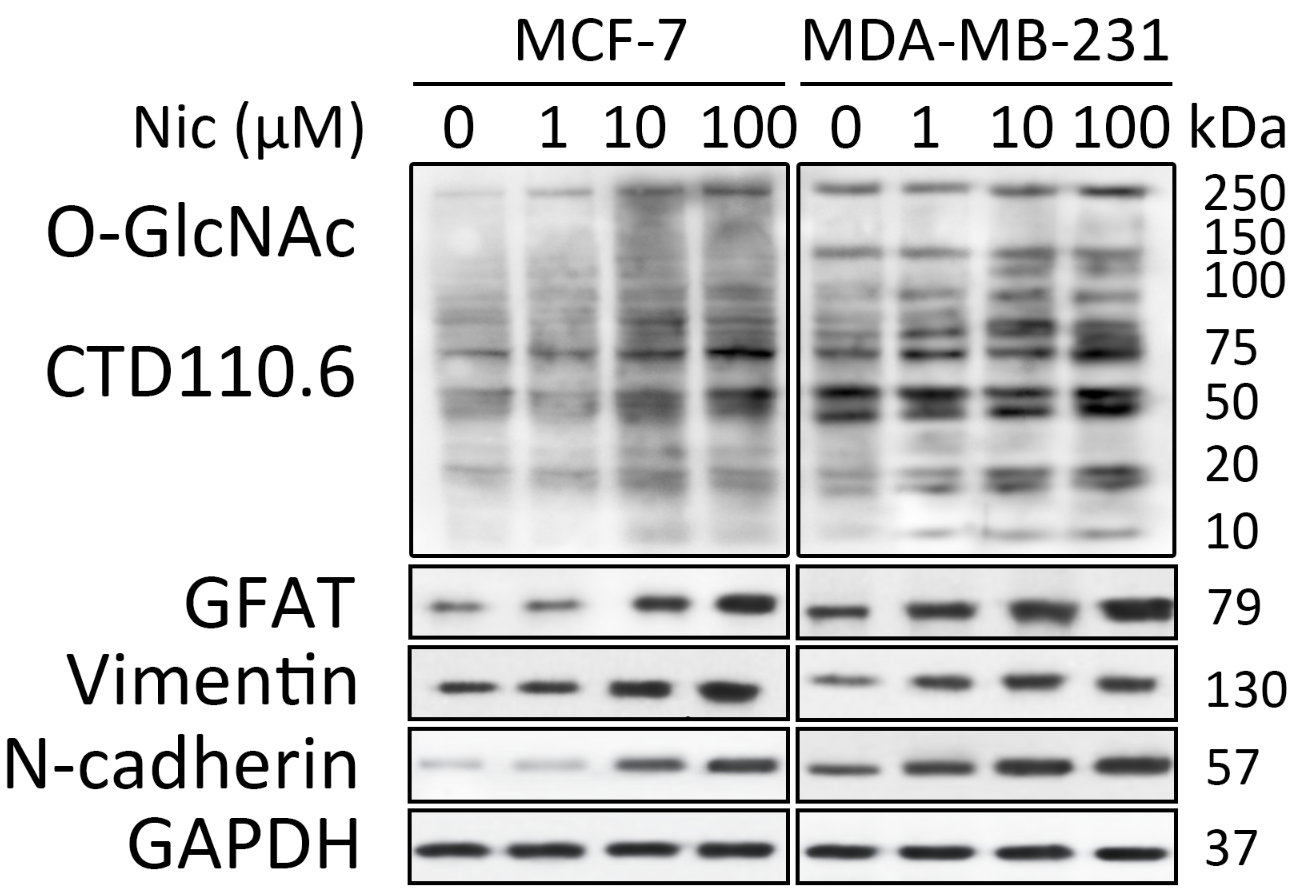


A


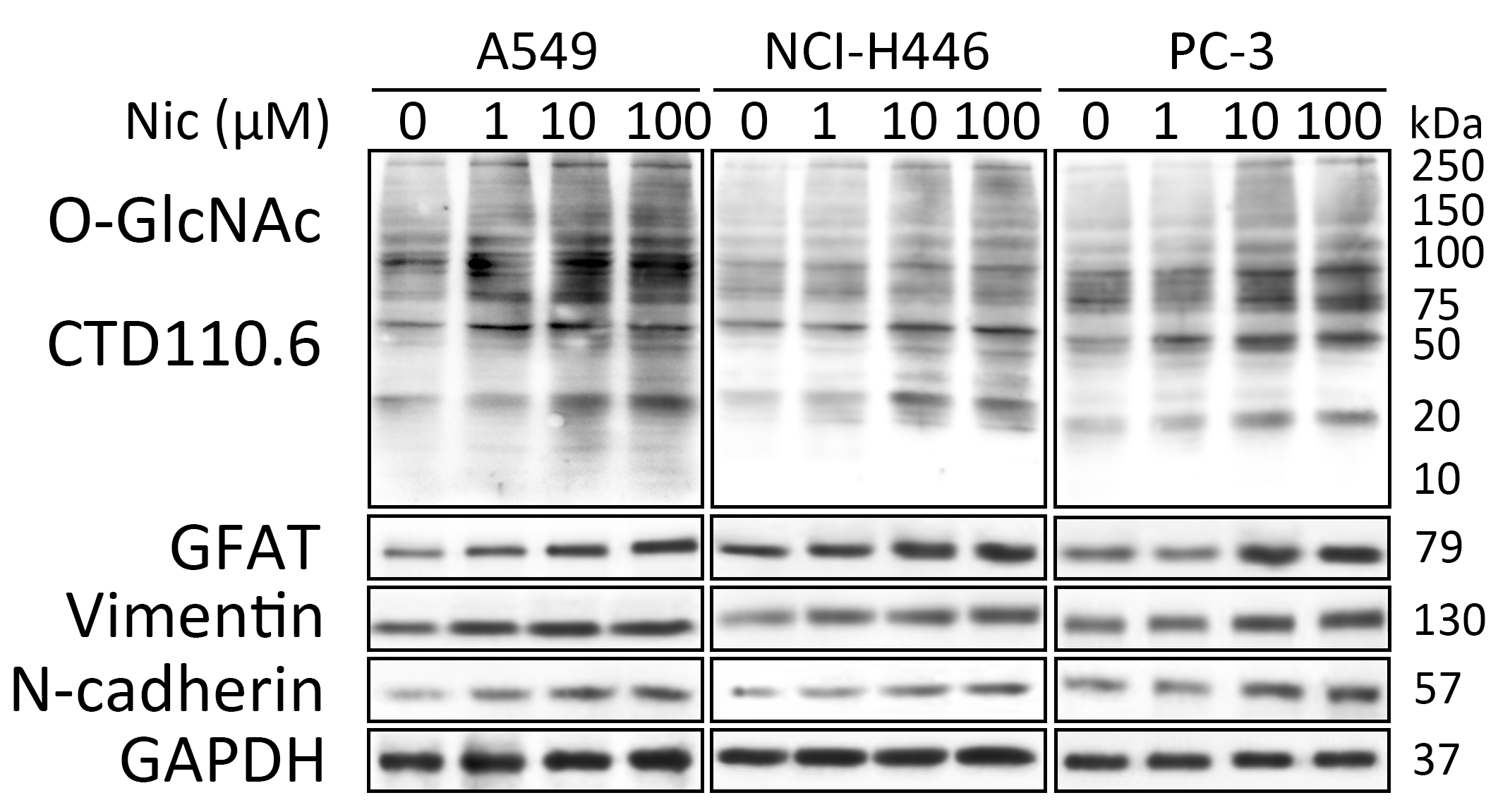

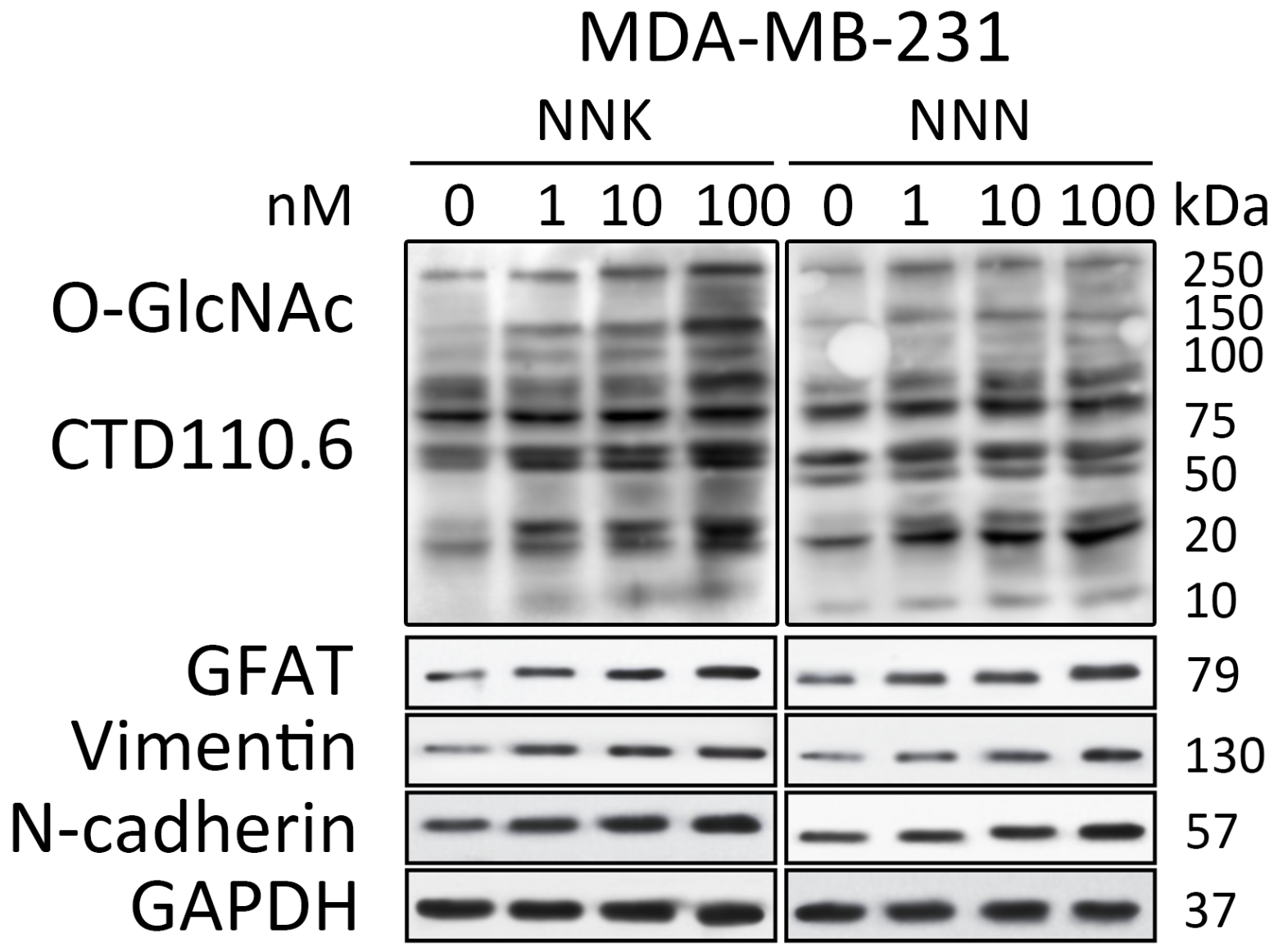


C

B


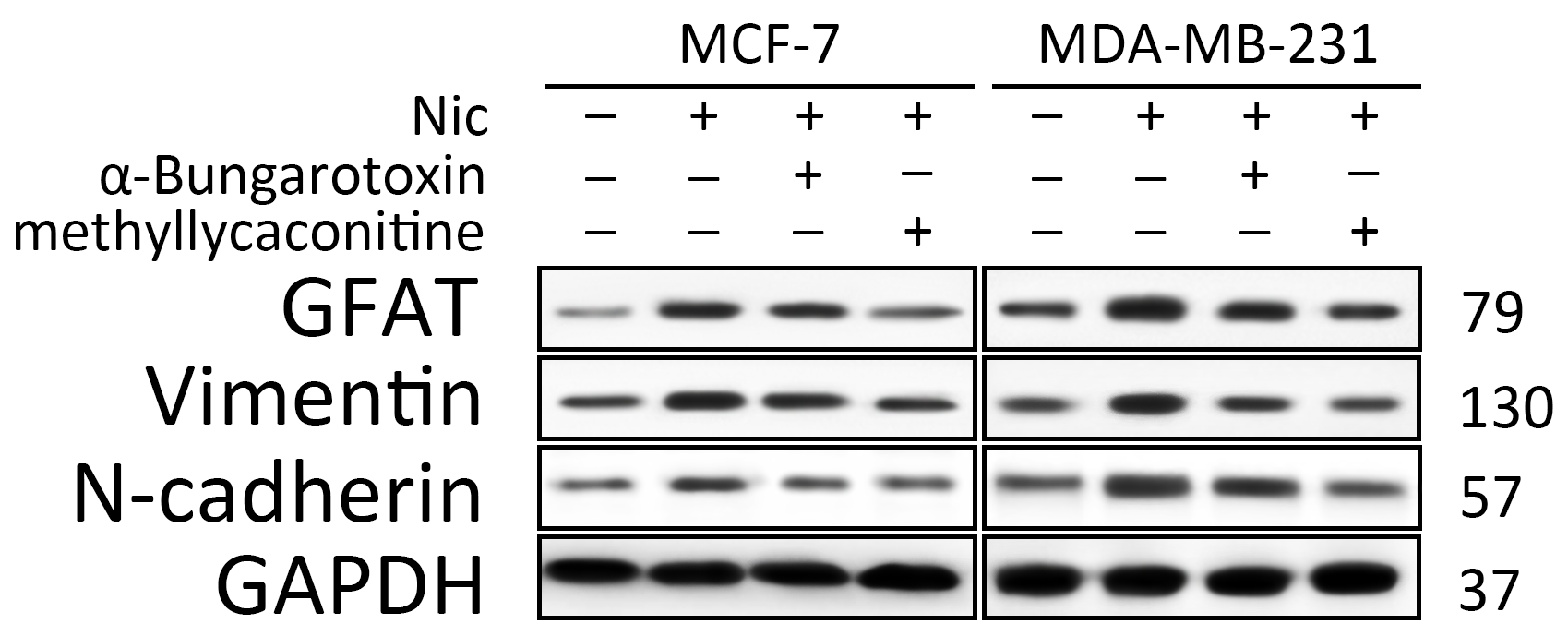


E

D

Fig. S1 (A) Breast cancer cells were treated with 1 μM, 10 μM or 100 μM Nic for 12 h. Cellular O-GlcNAcylation, expression of GFAT and EMT markers were analyzed by immunoblotting. GFAT transcript level was analyzed by quantitative RT-PCR. (B) A549 (non-small cell lung cancer cell line), NCI-H446 (small cell lung cancer cell line) and PC-3 (pancreatic cancer cell line) were treated with 1 μM, 10 μM or 100 μM Nic for 12 h. Cellular O-GlcNAcylation, expression of GFAT and EMT markers were analysed by immunoblotting analysis. (C) MDA-MB-231 cells were treated with 1 nM, 10 nM, 100 nM 4-(methylnitrosamino)-1-(3-pyridyl)-1-butanone (NNK) or N'-nitrosonornicotine (NNN) for 12 h. The cellular O-GlcNAcylation, expression of GFAT and EMT markers were analysed by immunoblotting analysis. (D) The nAChRs transcript level were analysed by quantitative RT-PCR in breast cancer cells. (E) To verify the role of nAChR The indicated cells were treated with 100 μM Nic with or without two nAChR antagonists (1 μM α-Bungarotoxin for α7-nAChR, 10 μM methyllycaconitine for α9-nAChR) for 12 h, respectively. The expression of GFAT and EMT markers were analysed by immunoblotting analysis. The data represent the means ± SEM, N = 3, **p* < 0.05, ***p* < 0.01.

Fig.S2

Fig. S2 O-GlcNAcylation regulates E-cadherin transcription in breast cancer cells with Nic treatment (100 μM, 24 h). Transfection of OGT siRNA or inhibition of O-GlcNAc (100 μM **L01**) increased E-cadherin transcription. The E-cadherin transcript level was analysed by quantitative RT-PCR. Scrambled siRNA was used as a control (CTRL). The data represent the means ± SEM, N = 3, **p* < 0.05, ***p* < 0.01.

Fig.S3

A


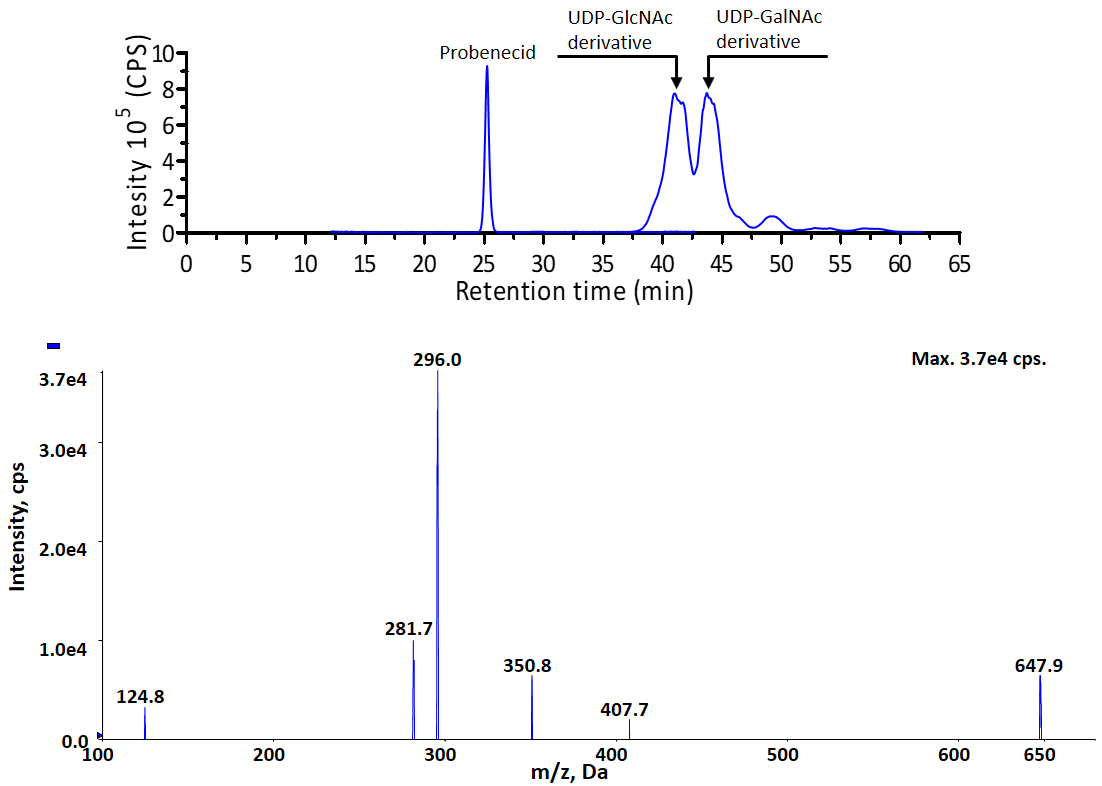


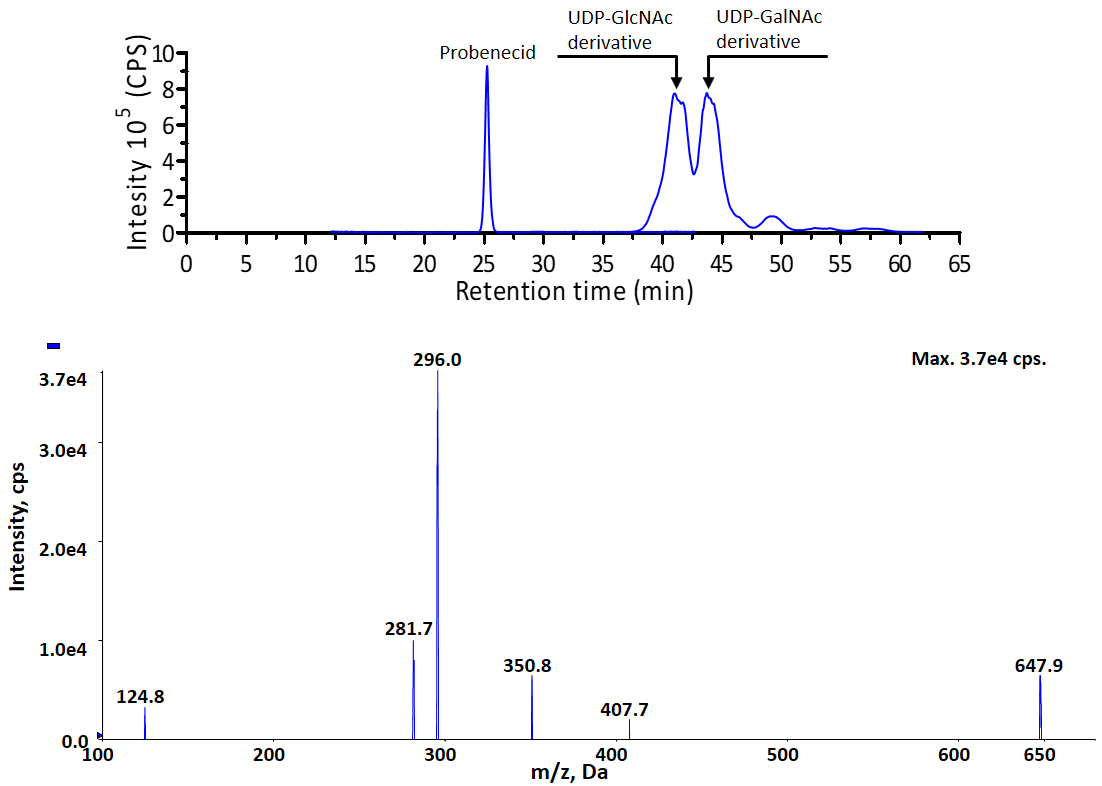


B


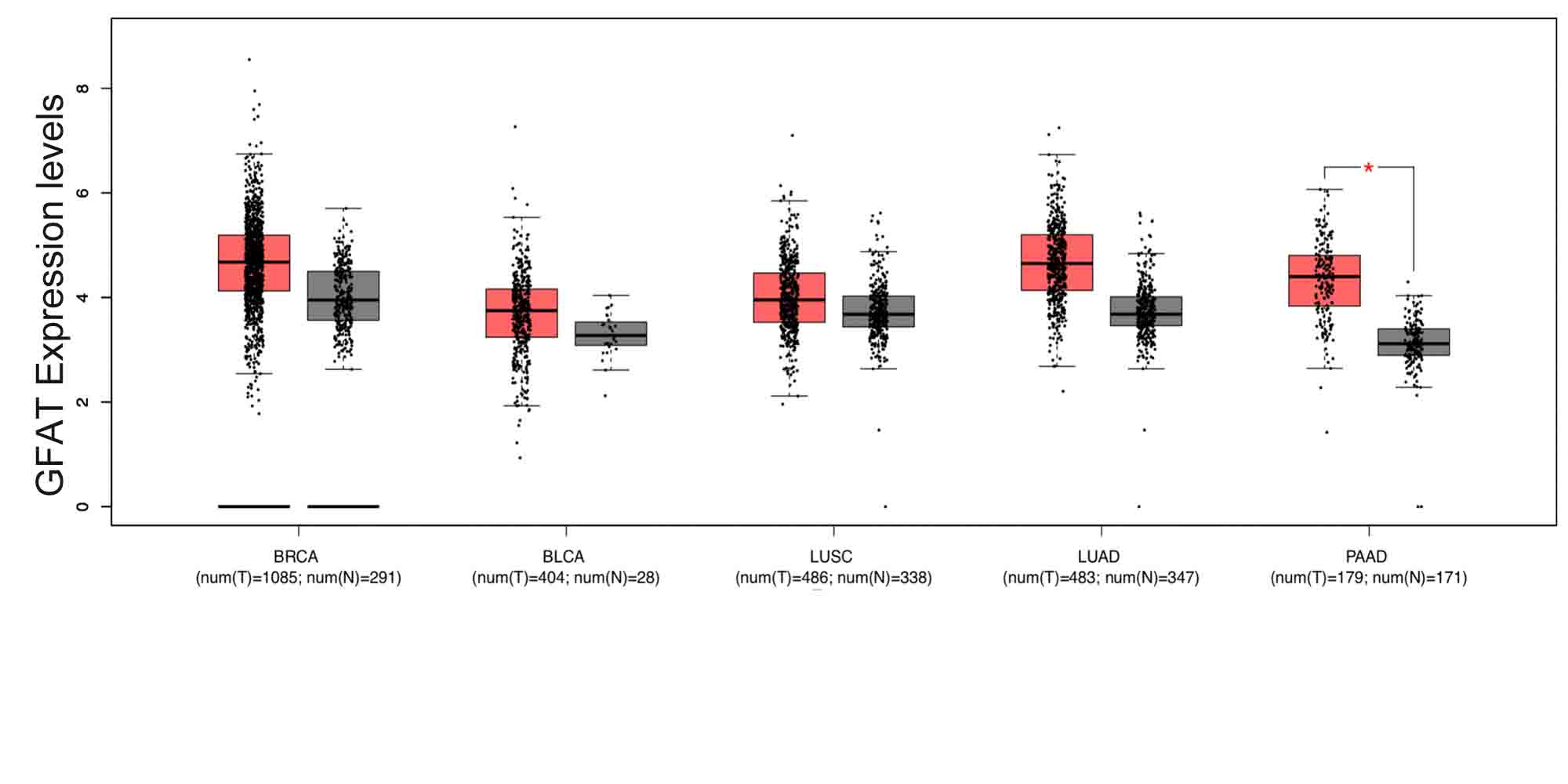


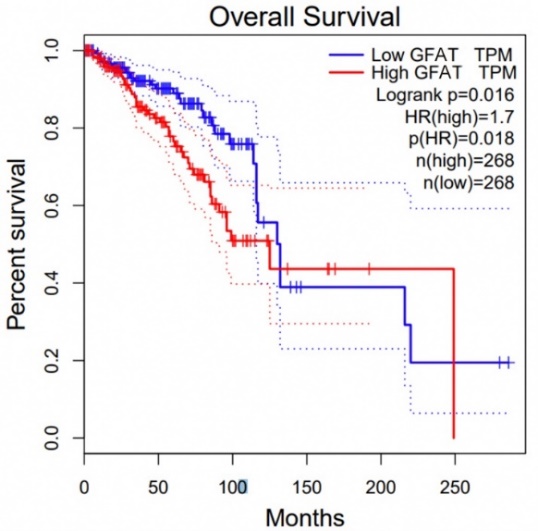


C

D

Fig. S3 (A) Separation of UDP-GlcNAc (10 μM), UDP-GalNAc (10 μM) and probenecid (10 μM) by using liquid chromatography. Mass spectrum of UDP-GlcNAc derivative peak from chromatogram of control cell extract. (B) GFAT was up-regulated in breast (BRCA) and other smoking related (BLCA, bladder urothelial carcinoma; LUSC, lung squamous cell carcinoma; LUAD, lung adenocarcinoma; PAAD, pancreatic adenocarcinoma) tumors (T) compared with the normal tissues (N). **p* < 0.05. (C) GFAT expression was negatively associated with overall survival of breast cancer patients. Data was from GEPIA database (<http://gepia.cancer-pku.cn/index.html>). (D) MCF-7 and MDA-MB-231 cells were treated with 100 μM Nic alone or together with 25 μM AZA for 24 h. The GFAT transcript level was analysed by quantitative RT-PCR.

Fig. S4


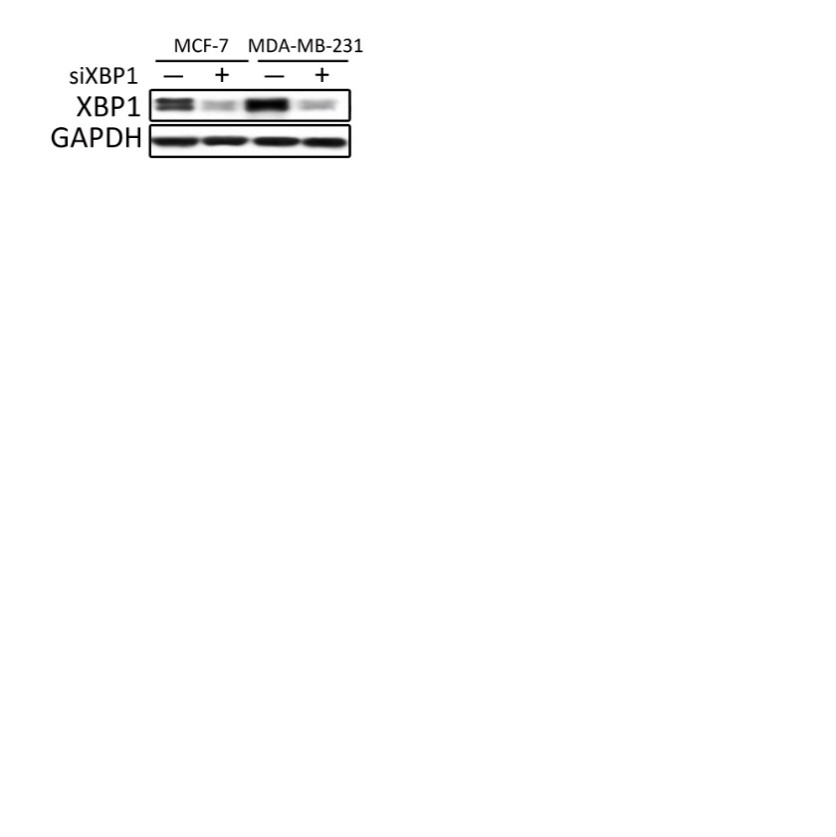


Fig. S4 XBP1 is not involved in GFAT transcription in Nic treated breast cancer cells. Indicated cells were transfected with XBP1 siRNA. The GFAT transcript level was analysed by quantitative RT-PCR. Scrambled siRNA was used as a control. The data represent the means ± SEM, N = 3, **p* < 0.05, ***p* < 0.01.

Fig.S5


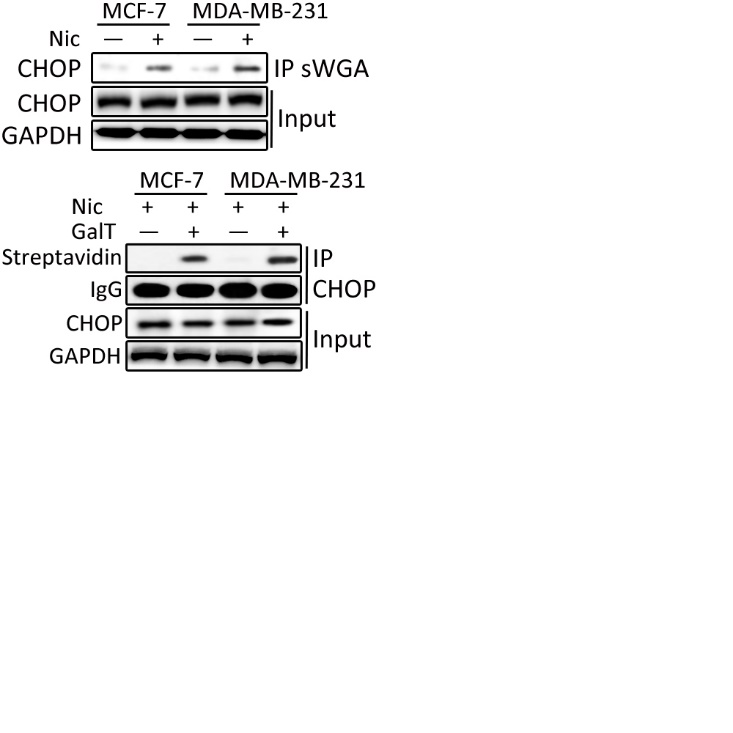


Fig. S5 MCF-7 and MDA-MB-231 cells were treated with 100 μM Nic for 24 h. SWGA affinity precipitation was performed, and precipitated fractions were analysed by immunoblotting for CHOP.

Fig.S6


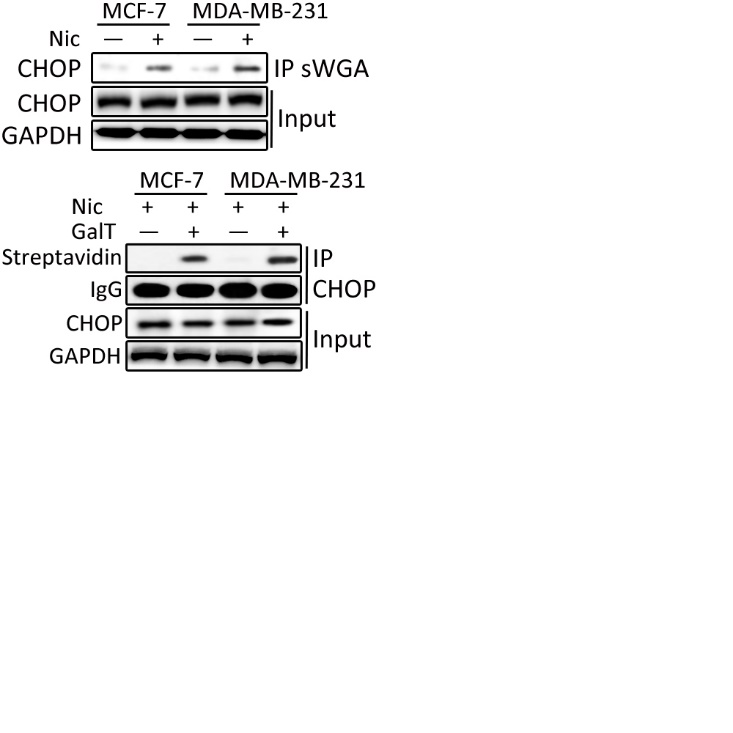


Fig. S6 Immunoblot were used to detected the O-GlcNAcylation of CHOP. Indicated cells were treated with 100 μM Nic for 24 h. Click-iT^™^ O-GlcNAc Enzymatic Labeling System was used. Biotin labled CHOP was detected by Streptavidin.

**References**

1. Liu, Y.*, et al.* O-GlcNAc elevation through activation of the hexosamine biosynthetic pathway enhances cancer cell chemoresistance. *Cell Death & Disease* **9**(5)**:** 485 (2018).

2. Zheng, J., Khalil, M. & Cannon, J. F. Glc7p Protein Phosphatase Inhibits Expression of Glutamine-Fructose-6-phosphate Transaminase from GFA1. *J Biol Chem* **275**(24)**:** 18070-18078 (2000).
